# Causal associations between gut microbiota and regional cortical structure: a Mendelian randomization study

**DOI:** 10.3389/fnins.2023.1296145

**Published:** 2023-12-22

**Authors:** Maochao Zhou, Song Chen, Yan Chen, Chunhua Wang, Chunmei Chen

**Affiliations:** ^1^Department of Neurosurgery, Fujian Medical University Union Hospital, Fuzhou, China; ^2^Fujian Institute of Neurosurgery, Fuzhou, China

**Keywords:** gut microbiota, brain cortical structure, Mendelian randomization study, gut-brain axis, neuropsychiatric disorders

## Abstract

**Introduction:**

Observational studies have reported associations between gut microbiota composition and central nervous system diseases. However, the potential causal relationships and underlying mechanisms remain unclear. Here, we applied Mendelian randomization (MR) to investigate the causal effects of gut microbiota on cortical surface area (SA) and thickness (TH) in the brain.

**Methods:**

We used genome-wide association study summary statistics of gut microbiota abundance in 18,340 individuals from the MiBioGen Consortium to identify genetic instruments for 196 gut microbial taxa. We then analyzed data from 56,761 individuals from the ENIGMA Consortium to examine associations of genetically predicted gut microbiota with alterations in cortical SA and TH globally and across 34 functional brain regions. Inverse-variance weighted analysis was used as the primary MR method, with MR Egger regression, MR-PRESSO, Cochran’s *Q* test, and leave-one-out analysis to assess heterogeneity and pleiotropy.

**Results:**

At the functional region level, genetically predicted higher abundance of class Mollicutes was associated with greater SA of the medial orbitofrontal cortex (*β* = 8.39 mm^2^, 95% CI: 3.08–13.70 mm^2^, *p* = 0.002), as was higher abundance of phylum Tenericutes (*β* = 8.39 mm^2^, 95% CI: 3.08–13.70 mm^2^, *p* = 0.002). Additionally, higher abundance of phylum Tenericutes was associated with greater SA of the lateral orbitofrontal cortex (*β* = 10.51 mm^2^, 95% CI: 3.24–17.79 mm^2^, *p* = 0.0046). No evidence of heterogeneity or pleiotropy was detected.

**Conclusion:**

Specific gut microbiota may causally influence cortical structure in brain regions involved in neuropsychiatric disorders. The findings provide evidence for a gut-brain axis influencing cortical development, particularly in the orbitofrontal cortex during adolescence.

## 1 Introduction

The gut microbiota, containing trillions of microbes, influences body function through critical pathways involving the enteric nervous system that connects to the central nervous system (Dupont et al., 2020). From an evolutionary perspective, the hologenome theory posits that genetic variation in the holobiont arises from both the host genome and microbiome (Cryan et al., 2019), underscoring the essential role of the gut microbiota in modulating brain function. 16S rRNA gene sequencing enables large-scale profiling of microbial abundances in a cost-effective manner. Accumulating evidence suggests gut dysbiosis contributes to various central nervous system conditions like Alzheimer’s, Parkinson’s, depression, and anxiety (Aho et al., 2019; Yang et al., 2020; Qian et al., 2021; Simpson et al., 2021).

The cerebral cortex, vital for cognitive abilities, can be measured *in vivo* using magnetic resonance imaging (MRI) to assess cortical surface area (SA) and thickness (TH) associated with neurological, psychological, and behavioral traits (Thompson et al., 2020). Hence, it’s feasible that using the cortical SA and TH as parameters for brain functions and structure alteration. Attention on the gut-brain axis, describing bidirectional signaling between the gut and brain (Cryan et al., 2019), has surged given demonstrations that the gut microbiota influences brain function through immune, endocrine, vagus nerve, and inflammatory pathways (Bravo et al., 2011; Cussotto et al., 2018; Matheoud et al., 2019).

MRI has provided an ideal approach for studying gut-brain interactions preclinically and clinically. A preclinical study using diffusion tensor imaging identified diet-dependent changes in white matter integrity associated with gut microbiome alterations in rats (Ong et al., 2018). Clinically, consuming fermented milk affected resting brain activity in healthy women, particularly in the periaqueductal gray, prefrontal cortex, basal ganglia, precuneus, and parahippocampal gyrus (Tillisch et al., 2013). Studies have uncovered links between irritable bowel syndrome, microbiota shifts, and brain changes (Labus et al., 2017; Pinto-Sanchez et al., 2017). A *Bifidobacterium longum* strain reduced amygdala and frontal-limbic responses to negative stimuli (Labus et al., 2017). Prevotella abundance associated with right hippocampal differences in emotional processing compared to Bacteroides (Pinto-Sanchez et al., 2017). Emerging evidence also connects the gut microbiota to neuropsychiatric disorders. One study linked schizophrenia prodrome to altered gut microbiota and elevated choline in the anterior cingulate cortex, potentially from increased short-chain fatty acid production (He et al., 2018). The gut microbiota also affects early brain development, with higher alpha diversity correlating to slower development but minimal impact on regional brain volumes at age two (Carlson et al., 2018).

**Figure 1:**
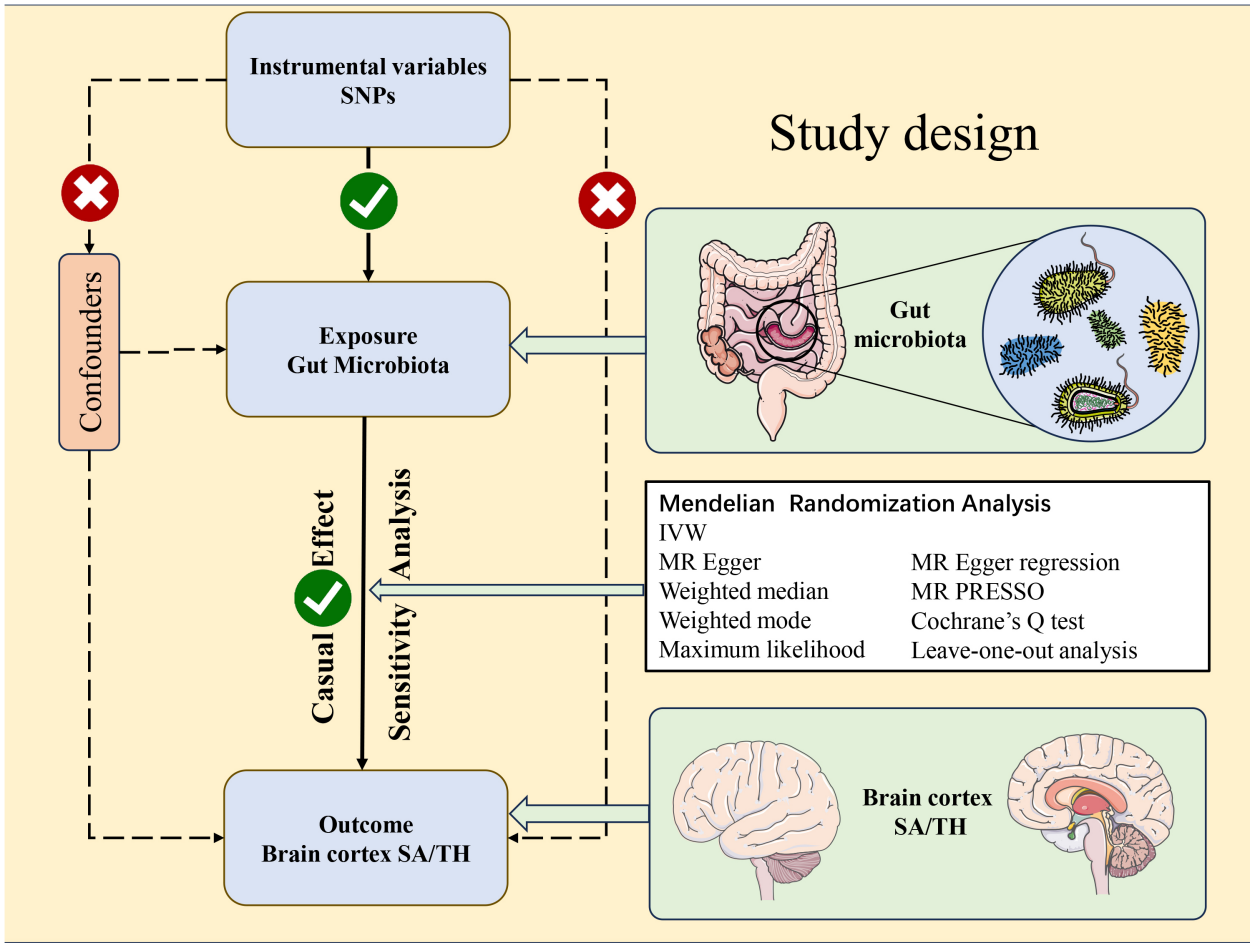
Study design of the Mendelian randomization study between gut microbiota taxa and the brain cortical structure as defined using magnetic resonance imaging-measured brain cortical surficial area and thickness.

While studies increasingly demonstrate the gut microbiota shapes brain structure and function, limitations exist due to insufficient statistical power from small samples, inconsistent interpretations, and confounders in observational studies. Mendelian randomization (MR) utilizes genetic variants as proxies for exposures, enabling investigation of causal relationships while controlling biases (Ong and Mac, 2019). Despite associations between the gut microbiota and brain structure (Tillisch et al., 2013; Labus et al., 2017; Pinto-Sanchez et al., 2017; Carlson et al., 2018; He et al., 2018), causality remains undetermined. Using large GWAS datasets, we conducted two-sample MR to explore the causal effect of the gut microbiota on cortical structure. We leveraged human genetics within the MR framework to elucidate the impact of the gut microbiota on SA and TH (Figure 1), including regional analyses. Our findings provide valuable insight into the gut-brain axis.

## 2 Method

### 2.1 Data source for gut microbiota

Summary statistics for gut microbiota were from a meta-analysis of GWAS in 18,340 multi-ethnic participants from the MinGen Consortium (23 European ancestry cohorts, >78% European ancestry, cohort details in Supplementary Table S11) (Kurilshikov et al., 2021). The genetic instrumental variables of each bacterial taxon were analyzed separately. The genome-wide association study of the gut microbiome adjusted for age, sex, technical factors, and principal components. After excluding unknown taxa (3 families, 12 genera), 196 instruments categorized by taxonomy were obtained - 9 phyla, 16 classes, 20 orders, 32 families, 119 genera - for Mendelian randomization.

### 2.2 Data source for brain cortex SA and TH

Brain cortical structure GWAS summary statistics were from the ENIGMA Consortium meta-analysis of MRI data from 51,655 individuals of predominantly European ancestry (60 global cohorts) (Grasby et al., 2020). Cortical TH and SA were measured. We utilized European ancestry results (cohort details in Supplementary Table S10). The Desikan-Killiany atlas parcellates the cortex into 34 regions bounded by gyral/sulcal anatomy (Desikan et al., 2006). Average TH and SA were measured for each region across hemispheres. We performed Mendelian randomization for the whole cortex and each region, analyzing 196 taxa per outcome. In total, 70 outcomes were obtained – global and regional TH and SA. Global analyses were weighted.

### 2.3 Selection of genetic instrument

Gut microbiota SNPs underwent the following quality control: (1) To obtain a sufficient number of SNPS, we chose a threshold of (*p* < 1 × 10–5) for SNP selection (Sanna et al., 2019). (2) Linkage disequilibrium pruning (LD *r*^2^ < 0.001, clump_kb = 10,000 bp) was performed in TwoSampleMR (v0.5.7) with EUR reference panel (Burgess and Thompson, 2011). (3) SNPs with F-statistic <10 were excluded to avoid weak instrument bias, calculated as (n-2) × R2/(1-R2) where R2 = 2 × MAF×(1-MAF) × β2. (4) Palindromic SNPs and those with intermediate allele frequencies were removed during harmonization. (5) SNPs reversing the overall direction in leave-one-out analysis were eliminated to ensure reliability.

We selected genetic instruments with *F* statistics >10 as robust variables. We verified results through Phenoscanncer to ensure confounders did not violate findings. We thoroughly assessed directional pleiotropy using MR-Egger intercepts and conducted leave-one-out analyses to evaluate SNP-driven bias. These steps aimed to achieve robust Mendelian randomization, meeting assumptions that: (i) instruments exhibit strong exposure associations; (ii) instruments are independent of confounders; and (iii) instruments affect outcomes only through the exposure.

### 2.4 Mendelian randomization analysis and sensitivity analysis

To assess the potential causal relationship between gut microbiota and brain cortex structure, we primarily utilized the inverse-variance weighted (IVW) Mendelian randomization method. The weighted median, MR-Egger regression, weighted mode, and maximum likelihood were also performed for validation and to corroborate IVW results.

To evaluate heterogeneity, we calculated Cochran’s *Q* statistic, performed MR-Egger intercept testing, and leave-one-out analysis to assess pleiotropy. The MR-PRESSO test was applied to detect and correct for outliers and heterogeneity when significant associations were identified.

To account for confounding, SNPs with significant MR estimates were assessed in PhenoScanner^1^ for associations with central nervous system disease risk factors (e.g., Alzheimer’s, depression, cerebrovascular events). Significant hits were excluded and MR re-run to evaluate robustness. Sample overlap between the exposure and outcome datasets was assessed using an online tool^2^ to evaluate bias and type I error rates (Burgess et al., 2016).

### 2.5 Statistical sets

Analyses were conducted in R v4.2.2 using TwoSampleMR v0.5.7. A global significance level of 0.05 was set. Bonferroni correction established value of *p* thresholds for each taxonomic level based on the number of taxa – phylum (*n* = 9): 0.05/9; class (*n* = 16): 0.05/16; order (*n* = 20): 0.05/20; family (*n* = 32): 0.05/32; genus (*n* = 119): 0.05/119. MR results below these thresholds were significant. Results between the threshold and 0.05 were nominally significant. An online tool^3^ estimated statistical power. Power ≥ 0.8 was considered sufficient, allowing rejection of ≥4/5 false null hypotheses (Fox and Mathers, 1997; Burgess, 2014).

## 3 Ethics statement

This study utilized publicly available deidentified data from participant studies that were approved by an ethical standards committee with respect to human experimentation. No separate ethical approval was required for this study.

## 4 Role of funding source

The funders had no role in study design, data collection, data analysis, interpretation, or writing of the report. The corresponding author had full access to all data in this study and was ultimately responsible for the decision to submit for publication.

## 5 Result

In brief, SNPs were selected to genetically predict gut microbiota’s causal effect on 34 functional brain areas, the number of SNP for each MR analysis is ranged from 26 to 67. The F statistics value for each genetic instrument was larger than 10, indicating strong instruments (Pierce et al., 2011).

**Figure 2:**
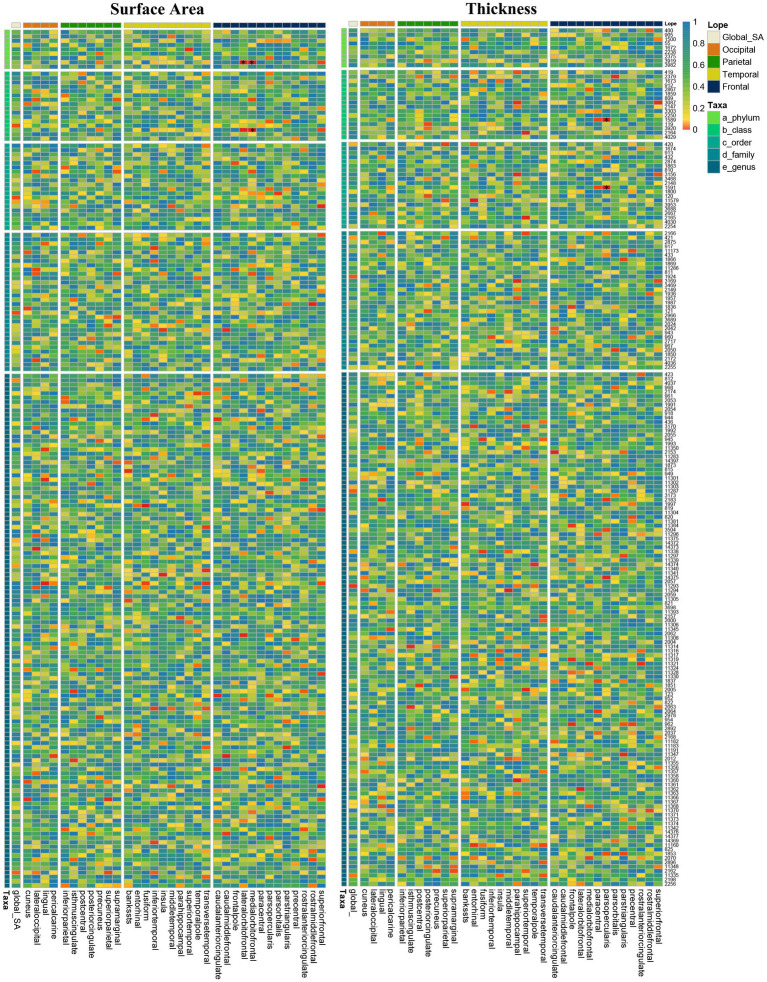
Heatmap of IVW estimates from196 gut microbiota taxa on global and 34 regions of brain cortical structure using MRI-measured surface area and thickness. The color of each cell represents the IVW-derived *p*-values of every MR analysis.

We conducted a comprehensive MR study examining the causal impact of genetically predicted 196 gut taxa on SA/TH of global and 34 brain functional areas. The heatmap showed IVW-derived *p-*values from all potential causal relationships between gut microbiota on brain cortex regions (Figure 2). Finally, we identified several significant or nominal significant gyrus influenced by gut microbiota. At the global level, four taxa, family Methanobacteriaceae, class Methanobacteria and order Methanobacteriales, were found to decrease SA and genus Turicibacter increased SA with a *p* value of IVW method <0.05 but not meeting Bonferroni corrected threshold. Another four taxa, genus familyXIIIAD3011group, family Defluviitaleaceae and Genus DefluviitaleaceaeUCG011 were found to increase TH and genus Tyzzerella3 decrease TH with p value of IVW method <0.05 but not meet Bonferroni corrected threshold. Gut microbiota had no causal relationship with the global SA and TH by strict value of *p* threshold.

**Figure 3:**
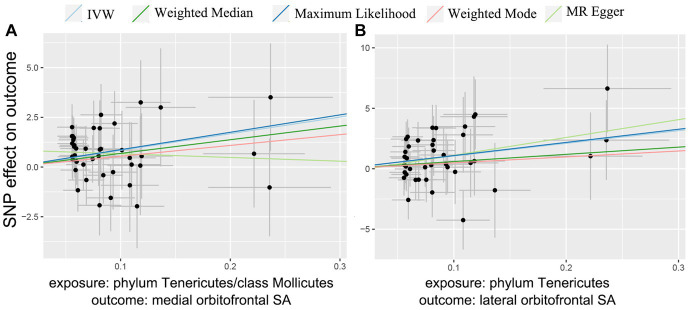
MR scatter plots of significant gut microbiota taxa on orbitofrontal SA.

At the brain functional region-level analysis, we found that 166 taxa had a value of p of the IVW method less than 0.05 on SA and 154 taxa on TH (Supplementary Tables S1–8). To be exact, three taxa, class Mollicutes on medial orbitofrontal (*β* = 8.39 mm^2^, 95% CI: 3.08–13.70 mm^2^, *p* = 0.0002), phylum Tenericutes on medial orbitofrontal (*β* = 8.39 mm^2^, 95% CI: 3.08–13.70 mm^2^, *p* = 0.0002) and phylum Tenericutes on lateral orbitofrontal (*β* = 10.51 mm^2^, 95% CI: 3.24–17.79 mm^2^, *p* = 0.0046), significantly increased SA (Table 1) (Supplementary Table S12). And the causal relationship trend of significant SA result is depicted as shown in the scatter plot (Figure 3). Also, two taxa, order Gastranaerophilales on parsopercularis (*β* = 0.0049 mm, 95% CI: 0.0017–0.0081 mm, *p* = 0.0016) and class Melainabacteria on parsopercularis (*β* = 0.0049 mm, 95% CI: 0.0017–0.0080 mm, *p* = 0.0016), significantly increase TH (Supplementary Table S13). And the scatter plot (Supplementary Figure S1) and forest plot (Supplementary Figure S2) shown the causal relationship trend of significant TH result. The rest of the taxa on specific gyrus had nominal significant *p* values. Given that the global TH of the brain cortex was 2.45 mm (SD = 0.11 mm) (Grasby et al., 2020), the alteration of gut microbiota causing changes in TH is not considered to reach clinically significant levels. No pleiotropy or heterogeneity was detected. Details are presented in the Supplementary data. To test whether the significant estimate was biased by risk factors, we conducted SNPs lookup in Phenoscanner (Supplementary Table S19). No significant biased SNPs were found.

**Table 1:** Significant Mendelian randomization estimates from gut microbiota on genetically predicted cortical structure in SA.

**Exposure**	**Outcome**	**IVW** ***P*-value**	***β* (95% Confidence intervals)**	**Cochran’s *Q*** ***P*-value**	**MR-Egger intercept** ***P*-value**	**N snp**
Class.Mollicutes.id.3920	Medialorbitofrontal SA	0.0020	8.39 mm^2^(3.08mm^2^-13.70mm^2^)	0.95	0.15	42
Phylum.Tenericutes.id.3919	Medialorbitofrontal SA	0.0020	8.39 mm^2^(3.08mm^2^-13.70mm^2^)	0.95	0.15	42
Phylum.Tenericutes.id.3919	Lateralorbitofrontal SA	0.0046	10.51 mm^2^(3.24mm^2^-17.79mm^2^)	0.79	0.66	40

Cochran’s *Q P*-value and MR-Egger intercept *P*-value < 0.05 is significant. IVW, Inverseb variance weighted; SA, cortical surficial area.

**Figure 4:**
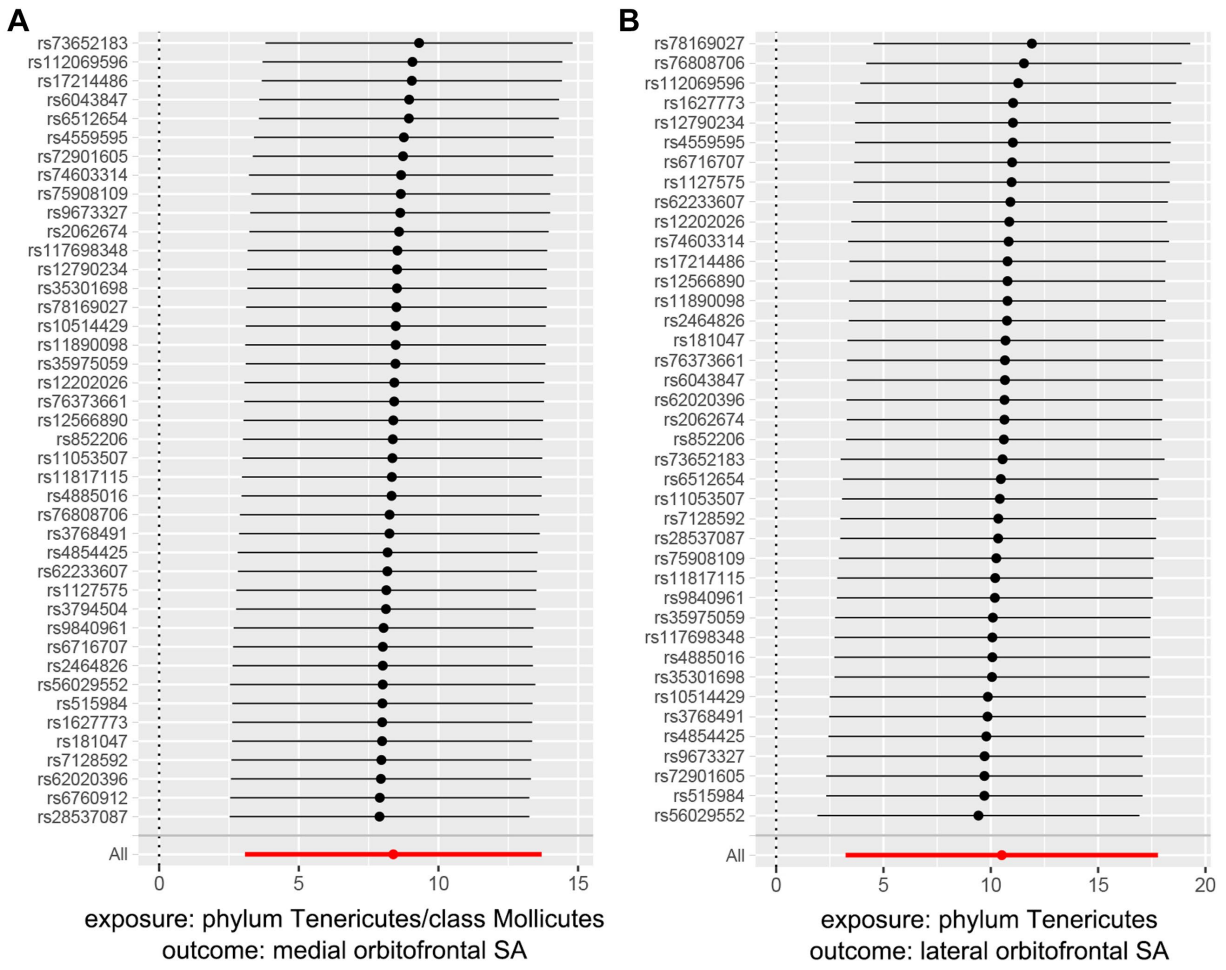
MR leave one out analysis plots of significant gut microbiota taxa on orbitofrontal SA.

For both statistically significant and nominally significant estimates, we employed Cochran’s *Q* test (Table 1), MR-Egger intercept test (Table 1), leave-one-out analyses (Figure 4 and Supplementary Figure S4), and funnel plots (Supplementary Figure S3) to evaluate horizontal pleiotropy (Supplementary Table S17). All *p*-values from the MR-Egger intercept tests and Cochran’s *Q* test were greater than 0.05, indicating no horizontal pleiotropy (Supplementary Table S18). MR-PRESSO results also found no heterogeneity (Supplementary Table S16). The estimates were not affected by individual single nucleotide polymorphisms (SNPs), indicating the robustness of the estimates. And power calculations shown the reliability of the results (Supplementary Table S14).

## 6 Discussion

To our knowledge, this is the first large-scale analysis that comprehensively investigates the causal relationship between gut microbiota and brain cortical structure using Mendelian randomization analysis. In this study, we systematically assessed the causal relationship between genetically predicted gut microbiota taxa and cortical structure across the brain. Our findings indicate that alterations in the abundance of specific microbial taxa can specifically impact cortical morphology, thereby providing causal evidence for earlier observational studies suggesting potential pathophysiological interactions between the gut microbiota and brain. These results substantiate and extend previous correlative research, emphasizing the presence of bidirectional signaling along the gut-brain axis.

Since the early 2000s, MRI has emerged as a prevalent technique for *in vivo* brain imaging. Consequently, it now presents an optimal approach for investigating gut-brain interactions within living organisms (Mayer et al., 2015). Previous observational studies have demonstrated that gut microbiota composition can alter brain structure and function. One study concluded the impact of fermented milk consumption on brain function in healthy women (Tillisch et al., 2013). Another two separate studies have proved the connection between irritable bowel syndrome (IBS), microbiota alterations, and related brain structure changes (Labus et al., 2017; Pinto-Sanchez et al., 2017). And studies have found that gut microbiota is associated with schizophrenia and affects children’s nervous system development (Carlson et al., 2018; He et al., 2018). These observational studies conclude an association between the gut microbiota and brain structure alteration detecting by MRI or neurological diseases, but cannot prove causality. Our study fills this limitation and provides a new perspective on the causal relationship between gut microbiota and cerebral cortex. As for the gut-brain axis causal findings, Crohn’s disease significantly decreased the TH of pars orbitalis. And IL-6 was observed to reduce the SA of middle temporal and increase the TH of fusiform and pars opercularis. Furthermore, a causal relationship between IL-6Rα and changes in the superior frontal and supramarginal (Liu et al., 2023). The brain structure alteration interacts cortical structure and Alzheimer’s disease, the study found that associations of the decreased SA of the temporal pole and decreased TH of cuneus with higher Alzheimer’s risk (Wu et al., 2021). These researches proved the casual relationship between inflammatory bowel disease or Alzheimer’s disease and changes in brain cortex. These results indicate that intestinal diseases and cerebral cortical changes, as well as cerebral cortical changes and brain function damage are cause-effect relationships. It established the link between intestinal diseases and brain diseases through cortical changes, which support our findings in this study. However, the above studies on causality are limited to the effects of specific diseases on functional areas, while this study focuses on a comprehensive summary of broad-spectrum gut microbiota and whole brain functional areas.

Overall, the gut microbiota has widespread effects on brain structure and function. However, the causal relationships between the gut microbiota and cortical structure or neuropsychiatric disorders have yet to be fully elucidated. Cerebral cortical SA and TH are regarded as neuroimaging biomarkers that can predict cognitive ability and higher-order brain functions (Desikan et al., 2006; Huang et al., 2023; Zhang et al., 2023). MRI can reveal causal associations between the gut microbiota and neuropsychiatric disorders, provided disease heterogeneity and severity are adequately addressed. Furthermore, MRI provides an objective method to evaluate the causal effects of the gut microbiota on SA and cortical TH alterations across the brain. Changes in brain structure may signify functional alterations and disease pathogenesis in neuropsychiatric disorders. In this study, we performed a comprehensive Mendelian randomization analysis to estimate the causal effects between gut microbiota taxa and cortical structural changes in the brain.

The main finding of our study is that orbitofrontal cortical SA is significantly influenced by the gut microbiota, including both the lateral and medial orbitofrontal regions. Moreover, the gut microbiota significantly affects cortical TH in the pars opercularis, although the small effect size limits potential clinical translation. The orbitofrontal cortex (OFC) plays a vital role in cognitive and behavioral regulation. OFC alterations have been associated with neuropsychiatric disorders including depression, schizophrenia, and addictive behaviors, as well as cognitive-motivational impairments (Olausson et al., 2007; Huang et al., 2023; Rompala et al., 2023; Wang et al., 2023). Furthermore, gut microbiota dysbiosis can increase gut permeability, thereby influencing hypothalamic–pituitary–adrenal (HPA) axis function and activity (Cussotto et al., 2018). The HPA axis regulates neurotransmitter release, brain-derived neurotrophic factor abundance, immune responses, and inflammation; all of which have been linked to depression pathogenesis (Du et al., 2020). Interestingly, the class Mollicutes belongs to the phylum Tenericutes. Research on these two microbial taxa is limited. However, studies have found Tenericutes and Mollicutes to be associated with intrahepatic cholestasis of pregnancy (Li et al., 2023), reduced risk of atopic dermatitis (Xue et al., 2023), and increased abundance in ulcerative colitis (Scaldaferri et al., 2023). Additionally, a positive correlation between Tenericutes and plasma leptin has been reported, which may relate to obesity pathophysiology (Cha et al., 2023).

Further investigations are warranted to elucidate the mechanisms underlying orbitofrontal cortex alterations and their associations with neuropsychiatric disorders. The role of pars opercularis cortical TH changes also merits exploration given this region’s importance for language and observations of structural alterations in multiple sclerosis (Cohen-Adad et al., 2011; Lindsay et al., 2020). Future studies should investigate whether gut microbiota-mediated changes in cortical SA or TH of the orbitofrontal and pars opercularis cortices influence brain function and contribute to the pathogenesis of disorders like depression and multiple sclerosis.

Estévez-López et al. retrospectively analyzed 12,286 prepubertal children and found that somatic symptoms were associated with reduced lateral orbitofrontal and parstriangularis cortical SA (Estévez-López et al., 2023). Since somatic complaints commonly manifest in depression, these findings further demonstrate that the gut microbiota may influence depressive disorder development by altering orbitofrontal cortex SA. Our estimates suggest the gut microbiota causally reduces orbitofrontal cortex SA. However, further research is needed to determine whether the gut microbiota contributes to depression by disrupting structural integrity of the orbitofrontal cortex.

**Figure 5:**
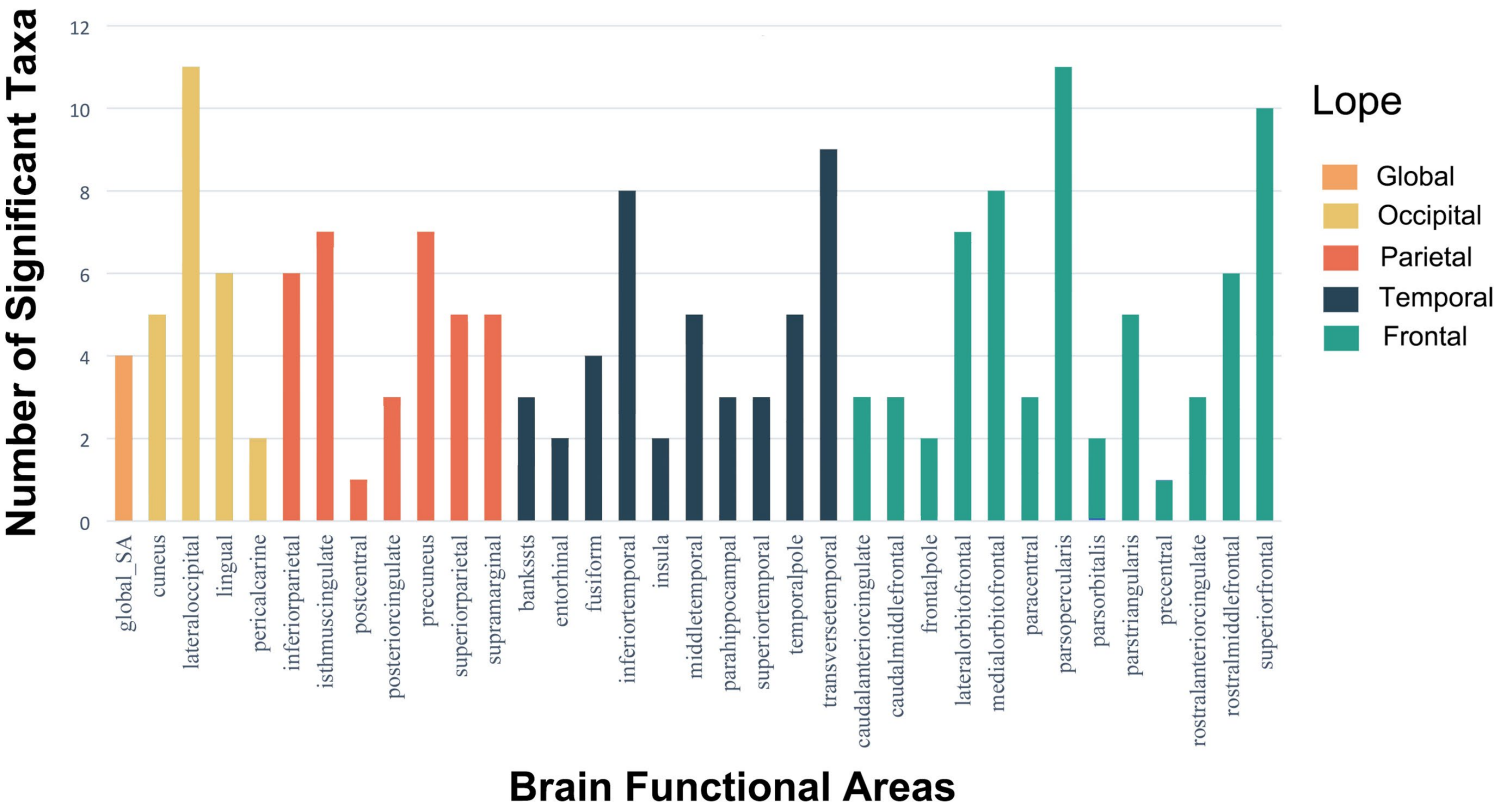
Histograms of the distribution of meaningful gut microbiota with *p*-values <0.05 in SA of global and 34 brain functional areas.

Although only five estimate passed Bonferroni correction in SA analysis, other estimates with IVW-derived *p* < 0.05 should also be interpreted cautiously. Figure 5 shows the gut microbiota significantly influenced the lateral occipital, parsopercularis, and superior frontal regions in SA based on microbiota taxa distribution across 34 brain regions at *p* < 0.05 (Supplementary Table S9). Previous research found a significant negative genetic correlation between total SA and average cortical TH (rG = −0.32, SE = 0.05, *p* = 6.50 × 10–12) (Chen et al., 2021). That study revealed SA was influenced by genetic variants altering neural progenitor cell gene regulation during fetal development, while active regulatory elements in the adult brain influenced TH. As the cerebral cortex develops continuously from fetal life through young adulthood, and the microbiota begins impacting fetal brain at birth, our results elucidate significant microbiota effects on SA, likely originating postnatally during cortical maturation. A growing number of preclinical studies demonstrate a consensus that gut microbes significantly impact brain structure/function in early life (Codagnone et al., 2019). This elucidates the microbiota’s effects on SA, implying influences on SA originate after birth through cortical maturation.

Numerous observational studies reveal microbiota-brain correlations, but we explored causal relationships between specific microbiota and structural (SA) and functional (TH) cortical changes across 34 regions. This provides new insights into the gut-brain axis concept.

Our study has several limitations. First, the enrolled patients were all European, so the causal relationship between gut microbiota and brain cortical structure in other populations remains unknown. Second, our findings only report alterations in the brain cortical structure of gut microbiota, but the underlying mechanisms warrant further investigation. Third, there is an overlap of 3 cohorts between the MiBioGen consortium and ENIGMA, with a total of less than 2,180 overlapped participants (11.89% overlap rate) in the MiBioGen consortium and 2,331 (4.11%) overlapped participants in the ENIGMA consortium. Given that it is difficult to achieve complete non-overlap summary data publicly. Our study’s proportion of overlapped participants is less than 11.89%. Based on Burgess’s simulation, the expected bias is less than 0.001, and the type I error is less than 0.05 (Supplementary Table S15). Four, the lack of heritability in gut microbiota GWAS data suggests that these associations may be part of a larger spectrum that was undetectable in the current GWAS sample size. This guarantees that future studies should utilize larger sample sizes, harmonized protocols, and more advanced approaches to microbiome analysis, including metagenomic sequencing, rather than 16S analysis and quantification of bacterial cell counts.

Our research results provide a new perspective to explore the causal relationship between gut microbiota and changes in SA and TH of the brain cortex, as well as the resulting neuropsychiatric disorders. The specific mechanisms through which gut microbiota influence changes in the brain cortex’s SA and TH require further investigation to explore the prevention and treatment of neuropsychiatric diseases resulting from these alterations.

## 7 Conclusion

This study is the first comprehensive MR analysis that reveals gut microbiota’s potential causal effect on the brain cortex structure, comprising 196 gut taxa and 34 functional brain regions. Our findings indicate that class Mollicutes and phylum Tenericutes causally increased SA of the orbitofrontal cortex. Clinical doctors must proceed with caution when encountering dysbiosis in a patient’s gut microbiota, especially when observing disturbances in the class Mollicutes and phylum Tenericutes.

## Data availability statement

The original contributions presented in the study are included in the article/Supplementary material, further inquiries can be directed to the corresponding author.

## Author contributions

MZ: Software, Validation, Writing – review & editing, Writing – original draft. SC: Formal analysis, Writing – original draft. YC: Conceptualization, Data curation, Investigation, Writing – review & editing. CW: Formal Analysis, Methodology, Validation, Writing – review & editing. CC: Conceptualization, Project administration, Validation, Writing – review & editing.

## Footnotes

^1^ http://www.phenoscanner.medschl.cam.ac.uk

^2^ https://sb452.shinyapps.io/overlap/

^3^ https://shiny.cnsgenomics.com/mRnd/
