# Supplementary figures and images for: Causal associations between gut microbiota and regional cortical structure: a Mendelian randomization study
Source: Front Neurosci. 2023 Dec 22;17:1296145. doi: 10.3389/fnins.2023.1296145 (PMC10774226; doi:10.3389/fnins.2023.1296145)

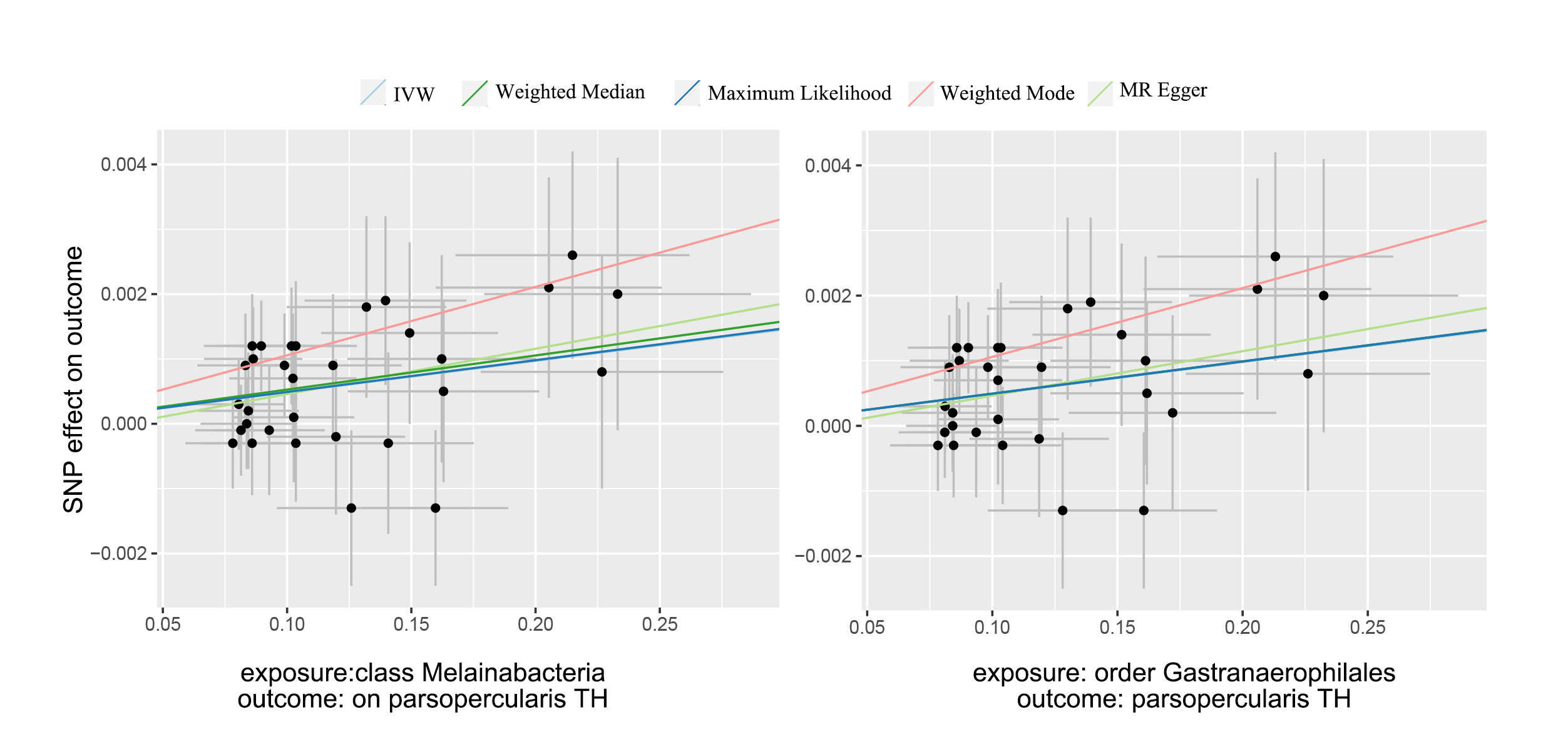

Supplement: Supplementary file 1 [file Image_1.jpg]

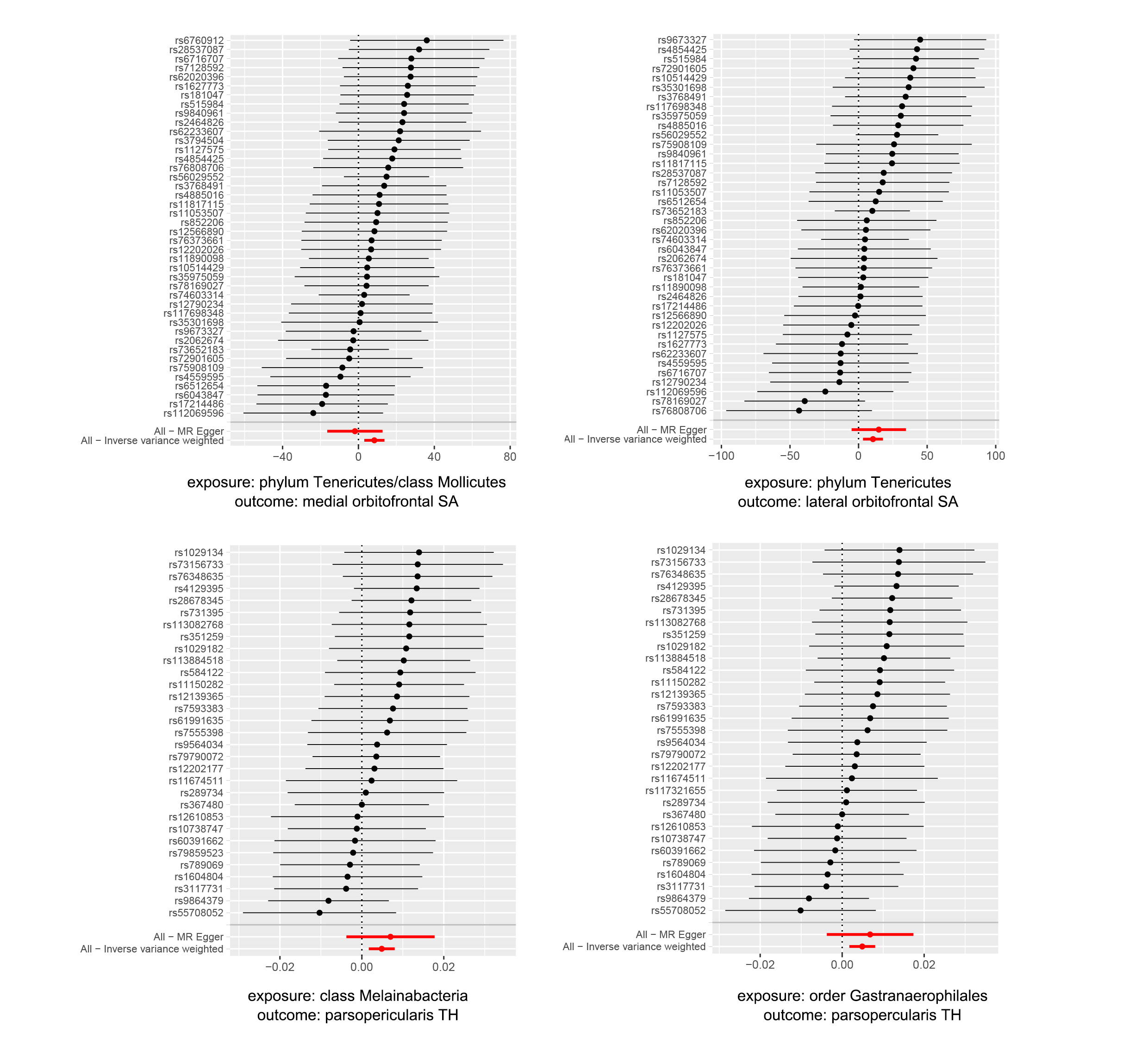

Supplement: Supplementary file 2 [file Image_2.jpg]

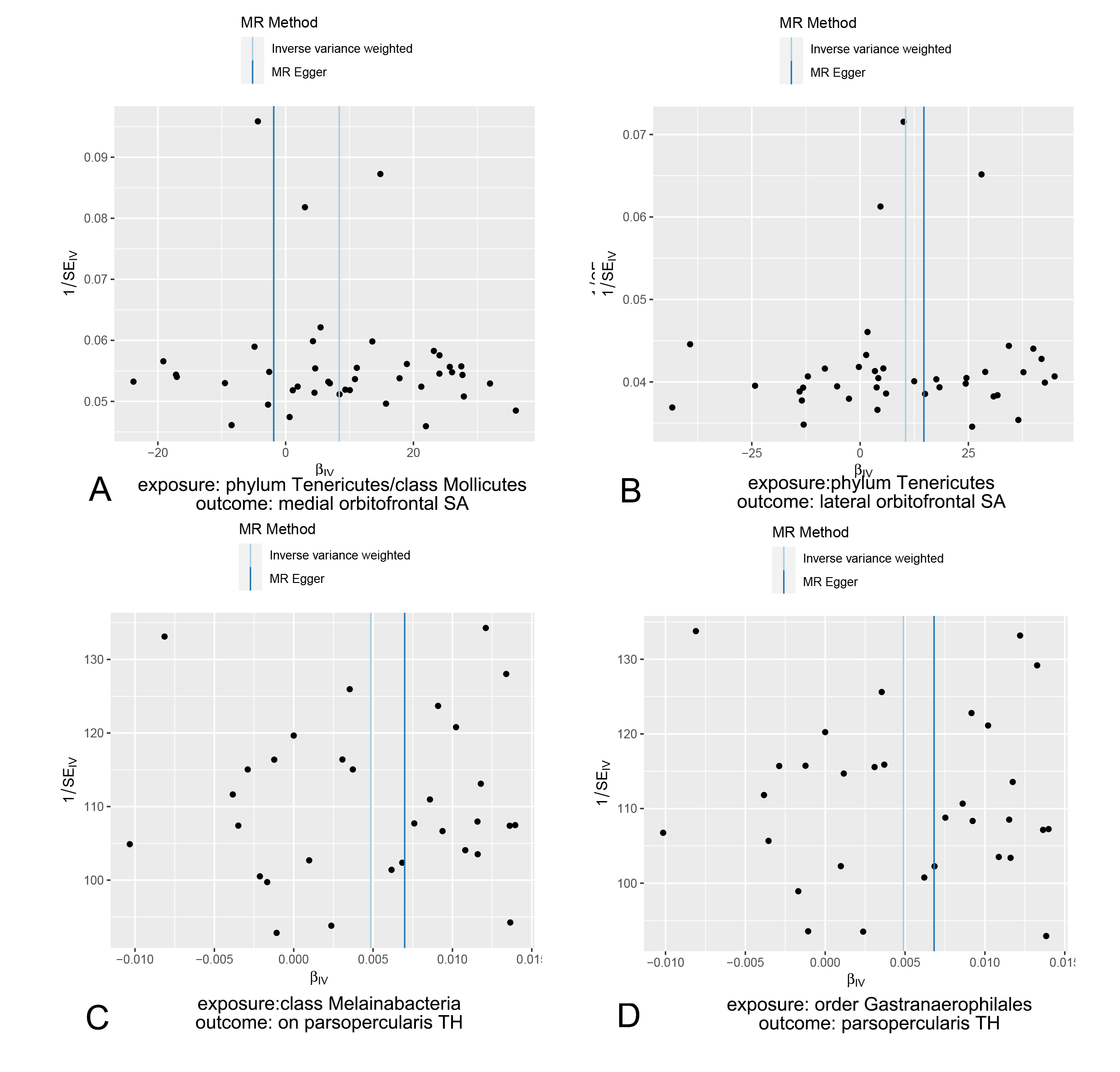

Supplement: Supplementary file 3 [file Image_3.jpg]

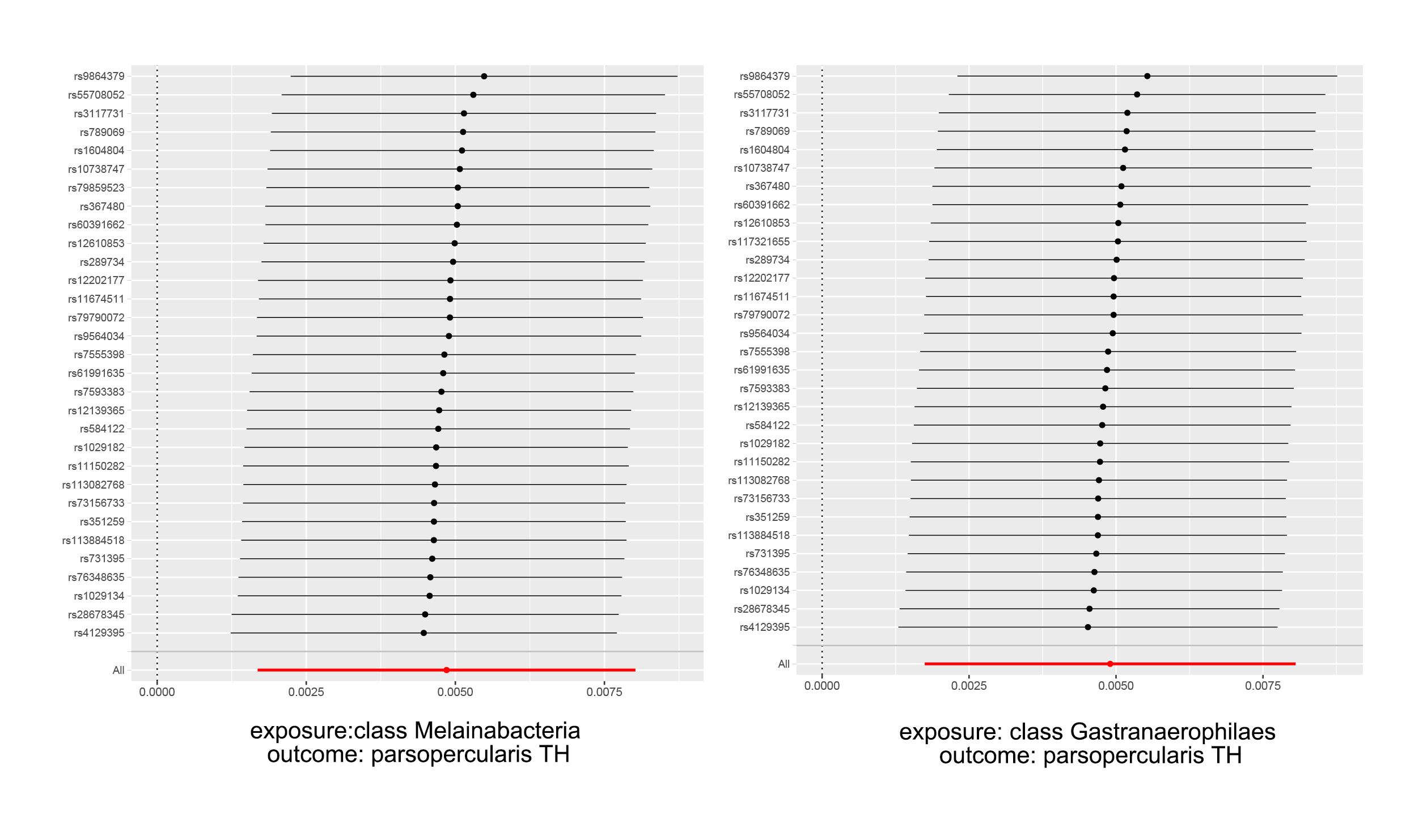

Supplement: Supplementary file 4 [file Image_4.jpg]
