# Supplementary material for: Causal associations between gut microbiota and regional cortical structure: a Mendelian randomization study
Source: Front Neurosci. 2023 Dec 22;17:1296145. doi: 10.3389/fnins.2023.1296145 (PMC10774226; doi:10.3389/fnins.2023.1296145)
Supplement: Supplementary file 6 [file Table_2.docx]

Table 1 Significant Mendelian randomization estimates from gut microbiota on genetically predicted cortical structure in SA.

| **exposure** | **outcome** | **IVW  P value** | **β (95% Confidence intervals)** | **Cochran’s Q  P value** | **MR-Egger intercept  P value** | **N snp** |
| --- | --- | --- | --- | --- | --- | --- |
| class.Mollicutes.id.3920 | medialorbitofrontal SA | 0.0020 | 8.39mm²(3.08mm²-13.70mm²) | 0.95 | 0.15 | 42 |
| phylum.Tenericutes.id.3919 | medialorbitofrontal SA | 0.0020 | 8.39mm²(3.08mm²-13.70mm²) | 0.95 | 0.15 | 42 |
| phylum.Tenericutes.id.3919 | lateralorbitofrontal SA | 0.0046 | 10.51mm²(3.24mm²-17.79mm²) | 0.79 | 0.66 | 40 |

Cochran’s Q P value and MR-Egger intercept P value < 0.05 is significant. IVW, Inverseb variance weighted; SA, cortical surficial area.

Figure 1：Study design of the Mendelian randomization study between gut microbiota taxa and the brain cortical structure as defined using magnetic resonance imaging-measured brain cortical surficial area and thickness.

Figure 2：Heatmap of IVW estimates from196 gut microbiota taxa on global and 34 regions of brain cortical structure using MRI-measured surface area and thickness. The color of each cell represents the IVW-derived P-values of every MR analysis.

Figure 3：MR scatter plots of significant gut microbiota taxa on orbitofrontal SA.

Figure 4：MR leave one out analysis plots of significant gut microbiota taxa on orbitofrontal SA.

Figure 5：Histograms of the distribution of meaningful gut microbiota with P values < 0.05 in SA of global and 34 brain functional areas.

Figure S1：MR scatter plots of significant gut microbiota taxa on parsopercularis TH.

Figure S2：MR Forest plots of significant gut microbiota taxa on SA and TH.

Figure S3：MR Funnel plots of significant gut microbiota taxa on SA and TH.

Figure S4: MR leave one out analysis plots of significant gut microbiota taxa on orbitofrontal TH.

Supplementary data: Original data and analysis result data.
